# Role of 5-HT1A Receptor in Vilazodone-Mediated Suppression of L-DOPA-Induced Dyskinesia and Increased Responsiveness to Cortical Input in Striatal Medium Spiny Neurons in an Animal Model of Parkinson’s Disease

**DOI:** 10.3390/molecules26195790

**Published:** 2021-09-24

**Authors:** Feras Altwal, Fernando E. Padovan-Neto, Alexandra Ritger, Heinz Steiner, Anthony R. West

**Affiliations:** 1 Center for Neurodegenerative Disease & Therapeutics, Rosalind Franklin University of Medicine and Science, North Chicago, IL 60064, USA; feras.altwal@my.rfums.org (F.A.); anthony.west@rosalindfranklin.edu (A.R.W.); 2 School of Graduate and Postdoctoral Studies, Rosalind Franklin University of Medicine and Science, North Chicago, IL 60064, USA; alexandra.ritger@my.rfums.org; 3 Discipline of Neuroscience, The Chicago Medical School, Rosalind Franklin University of Medicine and Science, North Chicago, IL 60064, USA; ferpadovan@usp.br; 4 Stanson Toshok Center for Brain Function and Repair, Rosalind Franklin University of Medicine and Science, North Chicago, IL 60064, USA; 5 Discipline of Cellular and Molecular Pharmacology, The Chicago Medical School, Rosalind Franklin University of Medicine and Science, North Chicago, IL 60064, USA

**Keywords:** dopamine, serotonin, L-DOPA, striatum, cortical stimulation, dyskinesia, Parkinson’s disease

## Abstract

L-DOPA therapy in Parkinson’s disease (PD) is limited due to emerging L-DOPA-induced dyskinesia. Research has identified abnormal dopamine release from serotonergic (5-HT) terminals contributing to this dyskinesia. Selective serotonin reuptake inhibitors (SSRIs) or 5-HT receptor (5-HTr) agonists can regulate 5-HT activity and attenuate dyskinesia, but they often also produce a loss of the antiparkinsonian efficacy of L-DOPA. We investigated vilazodone, a novel multimodal 5-HT agent with SSRI and 5-HTr_1A_ partial agonist properties, for its potential to reduce dyskinesia without interfering with the prokinetic effects of L-DOPA, and underlying mechanisms. We assessed vilazodone effects on L-DOPA-induced dyskinesia (abnormal involuntary movements, AIMs) and aberrant responsiveness to corticostriatal drive in striatal medium spiny neurons (MSNs) measured with in vivo single-unit extracellular recordings, in the 6-OHDA rat model of PD. Vilazodone (10 mg/kg) suppressed all subtypes (axial, limb, orolingual) of AIMs induced by L-DOPA (5 mg/kg) and the increase in MSN responsiveness to cortical stimulation (shorter spike onset latency). Both the antidyskinetic effects and reversal in MSN excitability by vilazodone were inhibited by the 5-HTr_1A_ antagonist WAY-100635, demonstrating a critical role for 5-HTr_1A_ in these vilazodone actions. Our results indicate that vilazodone may serve as an adjunct therapeutic for reducing dyskinesia in patients with PD.

## 1. Introduction

Parkinson’s disease (PD) is a devastating neurodegenerative disorder that is caused by a progressive loss of nigrostriatal dopamine (DA) neurons. Current therapeutic approaches typically focus on restoring central DA function, and treatment with the DA precursor levodopa (L-DOPA) remains the most effective pharmacological strategy to alleviate motor symptoms in PD [1,2,3]. However, long term L-DOPA treatment also produces debilitating motor side effects characterized by involuntary movements known as L-DOPA-induced dyskinesia [4,5,6]. In fact, the incidence of L-DOPA-induced dyskinesia is estimated to reach 90% after 10 years of treatment [7,8,9], which significantly reduces the therapeutic window and quality of life for patients with PD [10,11,12]. Thus, understanding the mechanisms underlying L-DOPA-induced dyskinesia is a critical step to improve L-DOPA therapy.

Several pathophysiological changes after DA denervation and chronic L-DOPA treatment have been identified that contribute to the development and expression of L-DOPA-induced dyskinesia. Many of these involve alterations in corticostriatal functional connectivity and dysregulation of striatal output (for reviews, see e.g., [13,14,15,16]). These cellular and molecular alterations include pre- and postsynaptic changes in the striatum [16]. For example, pronounced postsynaptic modifications occur in medium spiny projection neurons (MSNs), including DA receptor supersensitivity [17], which involves abnormally enhanced activation of intracellular signaling pathways, resulting in aberrant regulation of ion channels and receptors that produces abnormal responsiveness to corticostriatal inputs, as well as altered gene regulation and other effects (e.g., [14,18,19]).

Presynaptic changes associated with L-DOPA-induced dyskinesia include large fluctuations in striatal DA levels upon L-DOPA administration [2,20,21,22]. It is now accepted that these DA fluctuations drive postsynaptic changes in MSNs and L-DOPA-induced dyskinesia (e.g., [16]). These DA surges occur with severe loss of nigrostriatal DA neurons, and it is agreed that such L-DOPA-derived DA is released from other cells [16]. Of particular interest are the serotonergic (5-HT) fibers emanating from the dorsal raphe nucleus (DRN), which have the machinery to convert L-DOPA to DA and then release it [23,24]. These 5-HT fibers increase in density in the DA-depleted striatum (e.g., [25,26,27]). Further findings show that L-DOPA-derived DA released from 5-HT neurons is indeed critical for L-DOPA-induced dyskinesia ([21,22,28,29,30,31]; see [16,32], for recent reviews).

Attempts were made to pharmacologically regulate such abnormal DA release from 5-HT terminals and mitigate L-DOPA-induced dyskinesia by targeting the activity of 5-HT neurons in animal models [32,33]. For example, studies have investigated the use of selective 5-HT reuptake inhibitors (SSRIs), which block the 5-HT transporter [34,35,36,37], and agonists of the 5-HT_1A_ receptor (5-HTr_1A_), which stimulate 5-HT_1A_ autoreceptors on 5-HT neurons to attenuate their activity and abnormal DA release from their terminals (e.g., [30,37,38,39,40,41,42,43,44]). Results showed that many of these drugs significantly reduced dyskinesia scores in animal models. However, often these agents also diminished the prokinetic effects of L-DOPA, especially at higher doses, thus limiting their potential usefulness (e.g., [36,37,42,43,45]; see [32], for review). Clinical trials have so far been performed with 5-HTr_1A_ agonists [32], and these were typically less successful. For example, sarizotan, a 5-HTr_1A_ full agonist, showed promise in early preclinical [45,46] and clinical studies [47], but was ultimately not found to be superior to placebo in clinical trials [48].

Recent studies investigated a novel SSRI antidepressant, vilazodone, which was approved by the U.S. Food and Drug Administration (FDA) in 2011 [49], as a potential therapeutic to treat L-DOPA-induced dyskinesia. Vilazodone differs from previous agents in that it combines 5-HTr_1A_ partial agonist activity with its SSRI properties [50,51,52]. Early findings showed that vilazodone attenuated development and expression of L-DOPA-induced dyskinesia at doses that did not interfere with L-DOPA’s prokinetic efficacy [53,54]. Moreover, vilazodone also suppressed L-DOPA-induced aberrant gene regulation in striatal MSNs, a molecular correlate of L-DOPA-induced dyskinesia [54].

In the present study, we further investigated the impact of vilazodone on L-DOPA-induced dyskinesia and underlying mechanisms in the unilateral 6-hydroxydopamine (6-OHDA) rat model of PD. For one, we assessed vilazodone effects on the various subtypes of L-DOPA-induced dyskinesia (measured as “abnormal involuntary movements”, AIMs) in this model. Moreover, using in vivo electrophysiological techniques, we investigated the impact of vilazodone on the aberrant responsiveness to corticostriatal drive in striatal MSNs in the dyskinetic state. Furthermore, we determined whether these vilazodone effects were mediated by stimulation of 5-HTr_1A_. Our results show that vilazodone attenuated all types of L-DOPA-induced AIMs, as well as the increased MSN responsiveness to cortical drive, and that these effects were dependent on 5-HTr_1A_ activation.

## 2. Results

### 2.1. Evaluation of 6-OHDA Lesion

**Figure 1:**
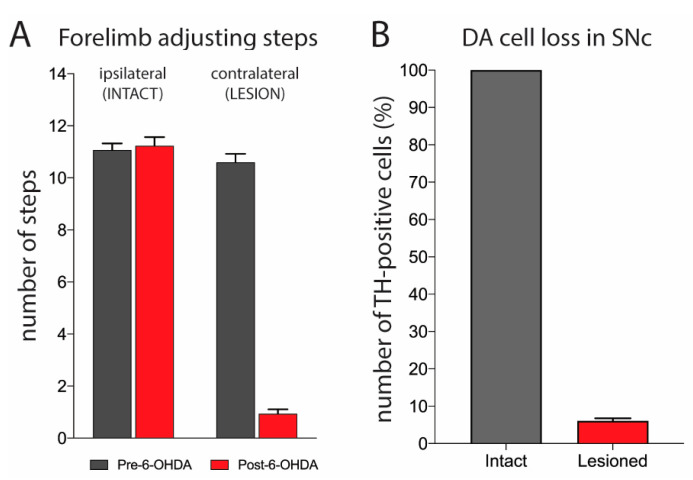
Unilateral DA lesion by 6-OHDA produces a contralateral forelimb stepping deficit. (**A**) Forelimb stepping scores (mean ± SEM) in tests performed pre- and four weeks post-surgery. These tests revealed that the 6-OHDA lesion produced a stepping deficit in the forelimb contralateral to the lesion, while stepping with the ipsilateral (intact) forelimb was not affected. The stepping scores given are from the included animals that showed three or fewer adjusting steps with the contralateral forelimb (n = 33). (**B**) Number of TH-positive cells in the SNc ipsilateral to the lesion, expressed as percentage of the TH-positive cells on the intact side. The included animals displayed a loss of 87.9–98.6% of TH-positive (DA) neurons on the side of the lesion (mean ± SEM, 93.95 ± 0.72% of intact side).

Stepping tests were performed before and after the 6-OHDA lesion to assess the impact of DA cell loss on forelimb movements. Only animals exhibiting a severe loss of forelimb movements, with a drop from a pre-surgery rate of approximately 11 adjusting steps with the forepaw contralateral to the lesion to three or fewer steps at 4 weeks post-surgery, were considered significantly impaired and were included in this study (Figure 1A). This approach has previously been shown to predict near-total DA lesions [55,56,57]. Tyrosine hydroxylase (TH) immunohistochemistry and cell counts were performed to confirm the lesions. The number of DA neurons in the substantia nigra pars compacta (SNc) ipsilateral (lesion) and contralateral (intact) to the side of 6-OHDA infusion was determined. Our results show that rats with three or fewer adjusting steps displayed a loss of DA neurons with a range of 87.9–98.6% (mean ± SEM, 93.95 ± 0.72% of intact side; Figure 1B).

### 2.2. Vilazodone Suppresses Established AIMs, but Does Not Affect Improved Stepping Performance, after L-DOPA Treatment

**Figure 2:**
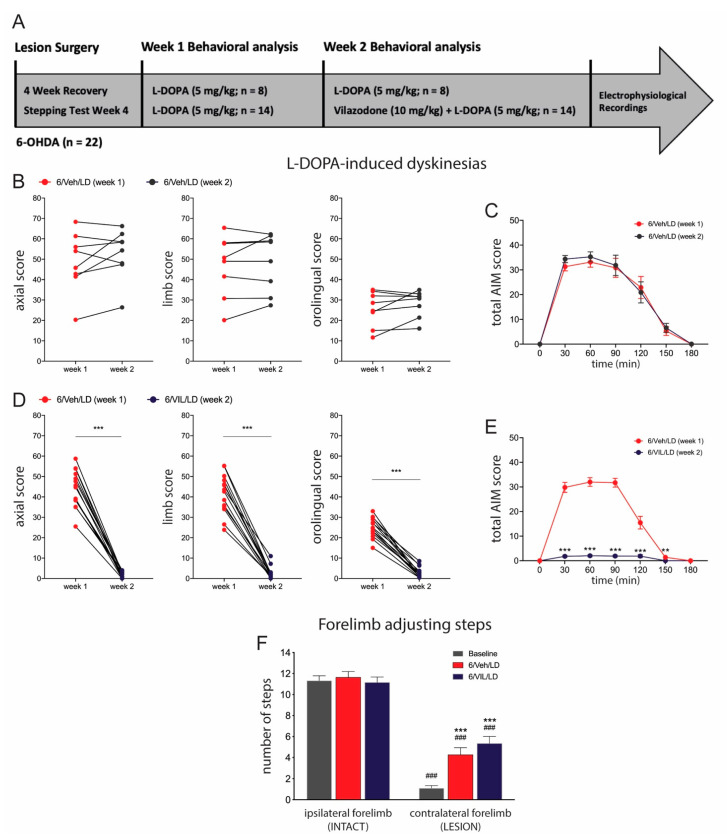
Vilazodone significantly inhibits expression of established AIMs, while not affecting the prokinetic effects of L-DOPA. (**A**) Experimental design/timeline of study. (**B**–**E**) AIMs in 6-OHDA-lesioned rats treated with vehicle (10% cremophor in 0.9% saline) + L-DOPA (5 mg/kg, i.p.) (6/Veh/LD) for two weeks (**B**,**C**), and in 6-OHDA-lesioned rats treated with vehicle + L-DOPA (6/Veh/LD) in week 1 followed by vilazodone (10 mg/kg, i.p.) + L-DOPA (6/VIL/LD) in week 2 (**D**,**E**). The video analysis revealed that vilazodone co-administration in week 2 significantly attenuated the expression of AIMs compared to L-DOPA-only treatment in week 1, for axial, limb and orolingual AIMs (individual animals) (**D**) and for total AIM scores (mean ± SEM) across 180 min after L-DOPA administration (**E**). *** *p* < 0.001, 6/VIL/LD (week 2) vs. 6/Veh/LD (week 1). (**F**) Forelimb stepping scores after these drug treatments. Scores (mean ± SEM) from 4 weeks after the lesion (before the start of the treatment protocol, “baseline”) and 1 h after L-DOPA administration on the second day of treatment week 2 are shown. The forelimb stepping test revealed that vilazodone, while inhibiting L-DOPA-induced AIMs, did not negate the prokinetic effects of L-DOPA in stepping behavior (6/VIL/LD vs. 6/Veh/LD, *p* > 0.05). ^###^ *p* < 0.001, vs. ipsilateral (INTACT) side; *** *p* < 0.001, vs. contralateral (LESION) baseline.

During the first week of drug treatment, all rats received L-DOPA (6/Veh/LD) and displayed axial, limb, and orolingual AIMs on the side of their body contralateral to the 6-OHDA lesion. These AIMs typically lasted for up to 3 h, with a peak in severity occurring around 30–90 min after L-DOPA administration (Figure 2). AIM subtypes were scored on the last three days of the week, and 3-day averages for each subtype and total scores are presented (Figure 2).

In week 2, rats received pretreatment with either vehicle or vilazodone 30 min prior to L-DOPA administration (Figure 2A). In vehicle-pretreated rats (6/Veh/LD, n = 8), AIM scores did not differ between week 2 and week 1, for axial, limb, orolingual, or total AIMs (all Z ≤ −1.26, *p* > 0.05; Wilcoxon matched-pairs signed-rank test) (Figure 2B,C). In contrast, vilazodone administration before L-DOPA (6/VIL/LD, n = 14) almost completely suppressed established axial, limb, orolingual, and total AIMs, compared to week 1 (all Z = −3.29, *p* < 0.001) (Figure 2D,E).

To evaluate drug effects on motor performance in these animals, we compared the number of forelimb adjusting steps before the start of the treatment protocol (i.e., 4-week counts, “baseline”) with stepping rates 1 h after L-DOPA administration on the second day (Tue) of treatment week 2 (Figure 2F). Two-factor ANOVA analysis revealed significant main effects of treatment (F(2,91) = 8.940, *p* < 0.001) and side (ipsilateral vs. contralateral to the lesion) (F(1,91) = 321.1, *p* < 0.001), with a significant interaction (F(2,91) = 8.798, *p* < 0.001). Tukey post-hoc tests revealed differences between groups as follows. Stepping with the forelimb contralateral to the 6-OHDA lesion was significantly reduced, compared to ipsilateral stepping, in all three conditions (*p* < 0.001). After L-DOPA-only treatment (6/Veh/LD), this motor performance was significantly improved (*p* < 0.001, vs. baseline). Similarly, rats that received vilazodone + L-DOPA co-treatment (6/VIL/LD) displayed a significant improvement in contralateral forelimb adjusting steps (*p* < 0.001, vs. baseline). These two groups did not differ in their contralateral steps (*p* > 0.05, 6/Veh/LD vs. 6/VIL/LD) (Figure 2F).

### 2.3. 5-HTr_1A_ Agonism Mediates Impact of Vilazodone on AIMs

**Figure 3:**
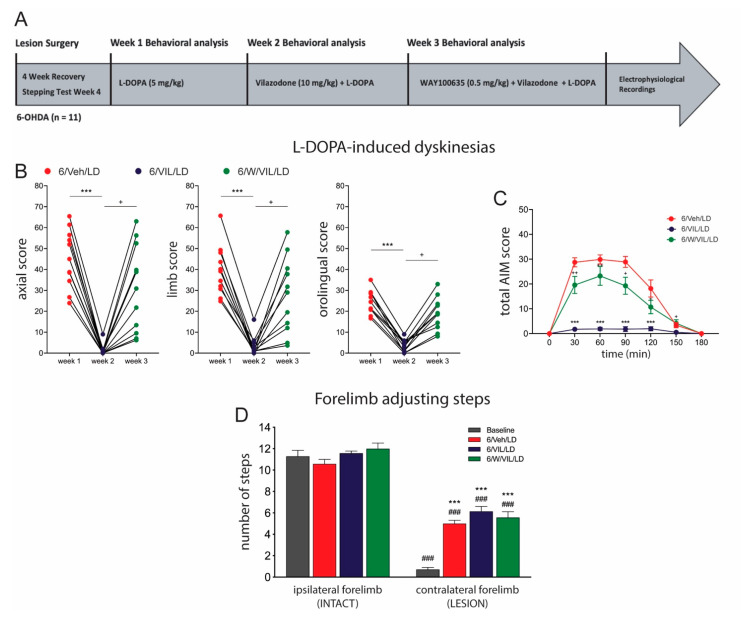
The 5-HTr_1A_ antagonist WAY-100635 (WAY) blocks the antidyskinetic effects of vilazodone. (**A**) Experimental design/timeline of study. (**B**,**C**) AIMs in 6-OHDA-lesioned rats treated with vehicle + L-DOPA (5 mg/kg, i.p.) (6/Veh/LD) in week 1, vilazodone (10 mg/kg, i.p.) + L-DOPA (6/VIL/LD) in week 2 and WAY-100635 (0.5 mg/kg, i.p.) + vilazodone + L-DOPA (6/W/VIL/LD) in week 3. The video analysis revealed that vilazodone co-administration (week 2) significantly attenuated the expression of AIMs compared to L-DOPA-only treatment (week 1), whereas WAY co-administration (week 3) significantly inhibited this beneficial effect of vilazodone treatment. This was shown for axial, limb and orolingual AIMs (individual animals) (**B**) and for total AIM scores (mean ± SEM) across 180 min after L-DOPA administration (**C**). *** *p* < 0.001, 6/VIL/LD vs. 6/Veh/LD; ^+^ *p* < 0.05, ^++^ *p* < 0.01, 6/W/VIL/LD vs. 6/VIL/LD. (**D**) Forelimb stepping scores (mean ± SEM) after these drug treatments. Neither vilazodone (6/VIL/LD) nor WAY (6/W/VIL/LD) had any effects on the L-DOPA-induced motor improvements (6/Veh/LD vs. baseline), as assessed by the forelimb stepping test. ^###^ *p* < 0.001, vs. ipsilateral (INTACT) side; *** *p* < 0.001, vs. contralateral (LESION) baseline).

In week 1 of our 5-HTr_1A_ antagonist experiment (Figure 3A), rats (*n* = 11) received repeated L-DOPA-only treatment (6/Veh/LD) and developed stable AIMs. Friedman ANOVAs showed a significant effect of treatments (all X^2^(2) > 18.73, *p* < 0.001). Dunn’s post-hoc tests revealed differences between treatment groups as follows. Consistent with our previous outcomes, vilazodone pretreatment (6/VIL/LD) in week 2 significantly reduced axial, limb, orolingual, and total AIMs compared to L-DOPA-only in week 1 (*p* < 0.001, vs. 6/Veh/LD) (Figure 3B,C). In contrast, administration of WAY-100635 (WAY), a selective 5-HTr_1A_ antagonist, together with vilazodone and L-DOPA (6/W/VIL/LD) in week 3 significantly attenuated the antidyskinetic efficacy of vilazodone (*p* < 0.05, vs. 6/VIL/LD; *p* > 0.05, vs. 6/Veh/LD) (Figure 3B,C).

Drug effects on motor performance in these animals were assessed with the forelimb stepping test. Two-factor ANOVA analysis of forelimb adjusting steps revealed significant main effects of treatment (F(3,48) = 19.57, *p* < 0.001) and side (ipsilateral vs. contralateral to the lesion) (F(1,48) = 541.1, *p* < 0.001), with a significant interaction (F(3,48) = 16.19, *p* < 0.001). Tukey post-hoc tests revealed the following differences between treatments. Stepping with the forelimb contralateral to the lesion was significantly reduced in all four conditions (*p* < 0.001). After treatment with L-DOPA-only (6/Veh/LD), vilazodone + L-DOPA (6/VIL/LD) or WAY + vilazodone + L-DOPA (6/W/VIL/LD), this stepping performance was significantly improved (*p* < 0.001, vs. baseline). Importantly, WAY administration (6/W/VIL/LD) did not alter this therapeutic efficacy as compared to L-DOPA-only treatment (*p* > 0.05, vs. 6/Veh/LD), or vilazodone + L-DOPA treatment (*p* > 0.05, vs. 6/VIL/LD) (Figure 3D).

### 2.4. Effects of Vilazodone and 5-HTr_1A_ Blockade on Striatal MSN Activity in Dyskinetic DA-Depleted Animals

**Figure 4:**
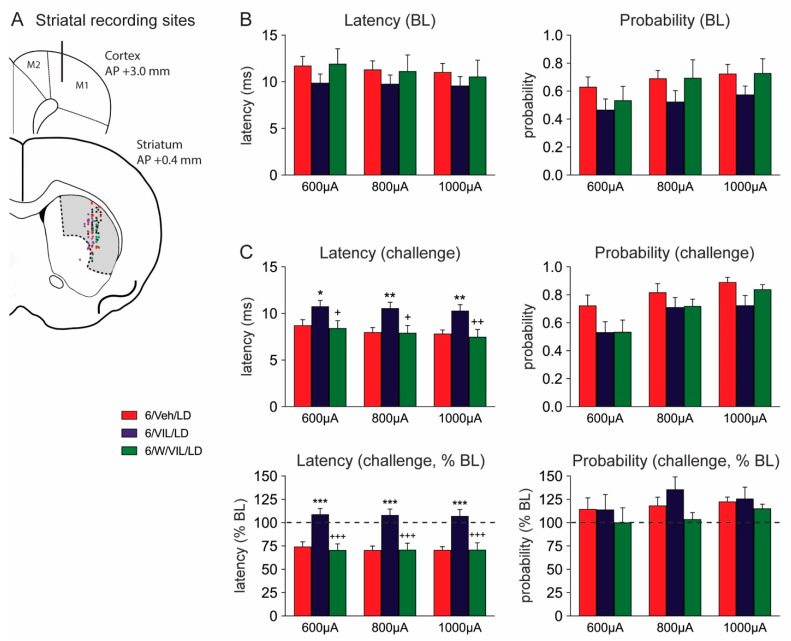
Vilazodone inhibits L-DOPA effects on spike onset latency after cortical stimulation in striatal MSNs, and this inhibition is mediated by 5-HTr_1A_ stimulation. (**A**) Schematic illustration of coronal sections through the frontal cortex and middle striatum (approximately at +3.0 and +0.4 mm rostral to bregma) showing the stimulation site in the primary motor cortex (M1), and, collapsed onto one level, the distribution of recording sites in the sensorimotor striatum (shaded) for the different treatment groups (“challenge” units: 6/Veh/LD, red dots; 6/VIL/LD, purple dots; 6/W/VIL/LD, green dots; pooled “baseline” units, black dots). (**B**) Cortically evoked responses in MSNs of 6-OHDA-lesioned rats recorded before the final drug treatment (“baseline” responses, BL). Displayed are spike onset latency (mean ± SEM) (left) and spike probability (right) in rats that had received a repeated pretreatment with vehicle + L-DOPA (5 mg/kg, i.p.) (6/Veh/LD; n = 11 cells in 5 rats), vilazodone (10 mg/kg, i.p.) + L-DOPA (6/VIL/LD; n = 14 cells in 8 rats), or WAY-100635 (0.5 mg/kg, i.p.) + vilazodone + L-DOPA (6/W/VIL/LD; n = 7 cells in 3 rats). (**C**) Cortically evoked responses in MSNs of 6-OHDA-lesioned rats recorded after the final drug treatment (“challenge” responses, top). Shown are spike onset latency (left) and spike probability (right) in rats that had received a repeated treatment with vehicle + L-DOPA (5 mg/kg, i.p.) (6/Veh/LD; n = 21 cells in 8 rats), vilazodone (10 mg/kg, i.p.) + L-DOPA (6/VIL/LD; n = 19 cells in 9 rats), or WAY-100635 (0.5 mg/kg, i.p.) + vilazodone + L-DOPA (6/W/VIL/LD; n = 16 cells in 7 rats). Additionally displayed are these data expressed in percentage of baseline values (% BL) (bottom). Vilazodone co-administered with L-DOPA (6/VIL/LD) prevented the L-DOPA-induced decrease in onset latency (6/Veh/LD). WAY administration (6/W/VIL/LD) blocked vilazodone’s ability to attenuate the L-DOPA effect on the onset latency. * *p* < 0.05, ** *p* < 0.01, *** *p* < 0.001, vs. 6/Veh/LD; ^+^ *p* < 0.05, ^++^ *p* < 0.01, ^+++^ *p* < 0.001, vs. 6/VIL/LD.

We used in vivo single-unit extracellular recordings to assess drug effects on cortically evoked activity in MSNs of the sensorimotor striatum ipsilateral to the 6-OHDA lesion (Figure 4). While the vast majority (≥95%) of striatal neurons are MSNs, we also infrequently encountered fast-spiking interneurons. These interneurons can be distinguished from MSNs by their short onset latency and duration of action potential responses to low stimulus intensities (<0.95 ms), and burst-like activity following cortical stimulation (see [58,59]). Only cells that exhibited an action potential duration of 1 ms or higher following cortical stimulation were included in this study. Tonically active interneurons typically did not respond to our stimulation protocol.

Cortical stimulation with 400 µA failed to elicit consistent responses in MSNs, and the 400 µA data were thus not included in the statistical analysis. For stimulation intensities of 600, 800 and 1000 µA, in cells recorded before drug administration (“baseline”; 6/Veh/LD, n = 11; 6/VIL/LD, n = 14; 6/W/VIL/LD, n = 7), two-factor ANOVAs showed the following results. Spike onset latency (Figure 4B, left) showed a tendency for a main effect of drug treatment (F(2,87) = 2.143, *p* = 0.12), no main effect of stimulation intensity (F(2,87) = 0.344, *p* > 0.05) and no significant interaction (F(4,87) = 0.053, *p* > 0.05). Analysis of spike probability (Figure 4B, right) revealed a significant main effect of drug treatment (F(2,87) = 3.976, *p* < 0.05), but no significant main effect of stimulation intensity (F(2,87) = 2.036, *p* > 0.05) and no significant interaction (F(4,87) = 0.132, *p* > 0.05). The 6/VIL/LD group showed tendencies for a reduction in onset latency and spike probability compared to the other groups; however, post-hoc tests did not reveal significant differences between individual groups (Figure 4B).

In cells recorded after drug administration (“challenge”; 6/Veh/LD, n = 21; 6/VIL/LD, n = 19; 6/W/VIL/LD, n = 16), two-factor ANOVAs of stimulation-evoked activity demonstrated the following. Spike onset latency (Figure 4C, top left) showed a significant main effect of treatment (F(2,159) = 15.49, *p* < 0.001), but no significant main effect of stimulation intensity (F(2,159) = 1.130, *p* > 0.05) and no significant interaction (F(4,159) = 0.064, *p* > 0.05). Analysis of spike probability (Figure 4C, top right) revealed significant main effects of treatment (F(2,159) = 4.938, *p* < 0.01) and stimulation intensity (F(2,159) = 9.112, *p* < 0.001), but no significant interaction (F(4,159) = 0.419, *p* > 0.05). Tukey post-hoc tests revealed the following differences between treatment groups (“challenge”; Figure 4C, top). Cells in vilazodone + L-DOPA-treated animals displayed a longer onset latency in cortically evoked spikes than cells in vehicle + L-DOPA-treated animals (6/VIL/LD vs. 6/Veh/LD) at 600, 800 and 1000 µA stimulation intensities (all *p* < 0.05) (Figure 4C, top left). Blocking 5-HTr_1A_ with WAY (0.5 mg/kg) prevented this effect of vilazodone on the onset latency, as WAY treatment significantly reduced the onset latency at all intensities (*p* < 0.05, 6/W/VIL/LD vs. 6/VIL/LD) to levels observed with L-DOPA-only treatment (*p* > 0.05, 6/W/VIL/LD vs. 6/Veh/LD). For spike probability (Figure 4C, top right), post-hoc tests did not show significant group differences at any of the 3 current intensities (*p* > 0.05).

Given the effects of the drug treatments on baseline activity (see above), we also expressed activities recorded after the drug challenge relative to baseline values (percent of baseline; Figure 4C, bottom). Two-factor ANOVAs for these data (“challenge”, % of baseline) revealed, for spike onset latency (Figure 4C, bottom left), a significant main effect of drug treatment (F(2,159) = 38.77, *p* < 0.001), no significant main effect of stimulation intensity (F(2,159) = 0.066, *p* > 0.05) and no significant interaction (F(4,159) = 0.041, *p* > 0.05). For spike probability (Figure 4C, bottom right), no significant main effects of treatment (F(2,159) = 1.949, *p* > 0.05), stimulation intensity (F(2,159) = 0.892, *p* > 0.05) or interaction (F(4,159) = 0.307, *p* > 0.05) were found. Tukey post-hoc tests (Figure 4C, bottom left) revealed that vilazodone + L-DOPA treatment produced a longer spike onset latency than vehicle + L-DOPA treatment (6/VIL/LD vs. 6/Veh/LD) at 600, 800 and 1000 µA stimulation intensities (all *p* < 0.001), and that blocking 5-HTr_1A_ with WAY completely prevented this effect of vilazodone (*p* < 0.001, 6/W/VIL/LD vs. 6/VIL/LD; *p* > 0.05, 6/W/VIL/LD vs. 6/Veh/LD).

In summary, consistent with previous findings demonstrating a significant increase in MSN activity in DA-depleted animals following L-DOPA treatment (e.g., [60,61,62]), our analysis showed that L-DOPA-only treatment (6/Veh/LD) reduced the MSN spike onset latency to 75% of baseline values. Vilazodone reversed this L-DOPA-induced facilitation of MSN responses, and this reversal was blocked by the 5-HTr_1A_ antagonist, WAY-100635.

## 3. Discussion

The present study investigated, in the 6-OHDA rat model of PD, the antidyskinetic effects of the multimodal serotonergic drug, vilazodone, the 5-HT receptor subtypes involved, and the electrophysiological correlates in the striatum of these drug actions. Our main results can be summarized as follows. First, our findings confirm and extend previous results by us [54] and others [53], demonstrating a powerful inhibitory effect of vilazodone on the various subtypes of L-DOPA-induced dyskinesia (AIMs) observed in this model. Second, importantly, in contrast to other serotonergic modulatory agents, vilazodone co-administration did not compromise the therapeutic efficacy of L-DOPA, as shown by our outcomes in the forelimb stepping test. Third, also in agreement with previous findings [53], these antidyskinetic effects of vilazodone were blocked by the selective 5-HTr_1A_ antagonist WAY-100635, demonstrating a critical role for 5-HTr_1A_ in this vilazodone action. Fourth, in line with the behavioral effects of vilazodone, our in vivo electrophysiological studies revealed that vilazodone prevented the abnormal L-DOPA-induced facilitation of corticostriatal transmission (reflected by a decrease in onset latency of cortically evoked spikes in MSNs), and that these vilazodone effects were also attenuated by blocking 5-HTr_1A_. These results complement our previous findings showing that vilazodone suppresses abnormal L-DOPA-induced gene regulation in MSNs in this model [54]. Collectively, these findings indicate that vilazodone co-treatment is capable of “normalizing” aberrant MSN activities and corticostriatal transmission that contribute to L-DOPA-induced dyskinesia, and that 5-HT_1A_ serotonin receptors mediate these vilazodone effects.

### 3.1. Characterization of Dopamine Lesion

The degree of DA cell loss after 6-OHDA infusion was determined by stereological quantification of the number of TH+ cells in the SNc. Our findings show that the DA-depleted animals that were included in this study, after meeting the inclusion criterion of three or fewer contralateral forelimb steps, had a near-total (average >93%) reduction in the TH+ cell numbers in the SNc ipsilateral to the 6-OHDA infusion. This is consistent with previous findings showing that rats with such a robust deficit in stepping performance had a 90% or greater loss of DA cell bodies in the SN [55,56], or an 80–100% loss of DA tissue content [63] or TH immunoreactivity [54,57] in the ipsilateral striatum.

### 3.2. Vilazodone Attenuates L-DOPA-Induced AIMs, but Does Not Block Prokinetic Effects of L-DOPA

In this study, as in previous studies (e.g., [54,57,64,65]), extensive 6-OHDA-induced striatal DA depletion, followed by repeated daily L-DOPA treatment, produced robust development and expression of AIMs, the rodent equivalent of L-DOPA-induced dyskinesia observed in patients with PD. Our recent work [54,57] demonstrated that L-DOPA given at the relatively low dose of 5 mg/kg once daily for 2–4 weeks was sufficient to induce AIMs in this PD model. Consistent with this finding, in the present study, AIMs emerged as early as one or two days after the first L-DOPA administration, and these AIMs stabilized during the last three days of week 1 and did not further increase between weeks 1 and 2.

A recent study [53] first demonstrated that vilazodone (10 mg/kg), when combined with L-DOPA, significantly reduced established AIMs in 6-OHDA-lesioned rats, an effect we confirmed for our model [54]. In agreement with these outcomes, we here report that vilazodone (10 mg/kg) pretreatment in week 2 almost completely abolished total AIMs as compared to week 1. Moreover, in this study, we provide a detailed analysis of the impact of vilazodone co-administration on the different AIM subtypes (i.e., axial, limb, and orolingual), in addition to the time course of AIM scores across 3 h after L-DOPA administration. Our results demonstrate that all AIM subtypes were dramatically suppressed, with the most robust inhibition seen for axial AIMs and a somewhat lesser effect for limb and orolingual AIMs.

Previous work that assessed SSRIs or 5-HTr_1A_ agonists as antidyskinetic agents found beneficial effects of those compounds, but also reported potentially problematic side effects, including 5-HT syndrome-like effects and a reduction in L-DOPA-induced motor improvement (e.g., [37,38,42,43,47,66]). In contrast, studies investigating vilazodone did not observe symptoms of 5-HT syndrome, even at higher doses [49,53,67]. Moreover, importantly, our findings demonstrate that vilazodone co-administration, at the present intermediate dose (10 mg/kg), which largely suppressed L-DOPA-induced abnormal gene regulation in striatal MSNs [54], did not interfere with the prokinetic efficacy of L-DOPA, as assessed in the forelimb stepping test (present results; [54]), although higher doses may lose some of this advantage [53].

The loss of the prokinetic effects of L-DOPA (e.g., [38,47]) has been attributed to a strong inhibition of 5-HT neurons innervating the striatum following a high-dose treatment with SSRIs or 5-HTr_1A_ full agonists, which might lead to a near-complete shutdown in striatal DA release from 5-HT terminals [68]. It can be speculated that the unique pharmacological profile of vilazodone as an SSRI together with its 5-HTr_1A_ partial agonist property, which is thought to desensitize 5-HTr_1A_ on the serotonergic cell bodies that regulate the firing activity of these neurons [68,69,70], may account for the efficacy seen with this multimodal agent. The impact of vilazodone may be sufficient to moderate serotonergic activity and DA release from these terminals, thus avoiding abnormal DA spikes in the striatum and their molecular [54] and behavioral (AIMs) consequences, without completely shutting down this DA input, and thus enabling prokinetic effects of L-DOPA.

### 3.3. Vilazodone Attenuates L-DOPA-Induced Facilitation of Corticostriatal Transmission in Dyskinetic Parkinsonian Rats

Hyperexcitability of MSNs has been described as a primary neuropathophysiological correlate of dyskinesia (e.g., [57,61,62,71,72]). In the DA-depleted striatum, DA receptors on MSNs become supersensitive, producing increased responsiveness of MSNs to dopaminergic drugs such as L-DOPA (e.g., [61,72]). This increase in MSN responsiveness after L-DOPA administration is most pronounced in the sensorimotor striatum [54,57,73,74]. We therefore assessed the effects of L-DOPA and vilazodone on cortical stimulation-evoked MSN activity in the sensorimotor striatum.

Consistent with previous work, our results show that chronic L-DOPA treatment produced an increase in MSN responsiveness to cortical stimulation, reflected by a decrease in spike onset latency. Enhanced responsiveness to corticostriatal drive after L-DOPA treatment has been related to aberrant hyperactivation of intracellular signaling pathways in MSNs (e.g., [44,71,72,75,76]). Importantly, in our study, this increase in MSN responsiveness was prevented when vilazodone was combined with L-DOPA treatment, a drug combination that also attenuated abnormal molecular signaling in MSNs [54]. Our findings suggest that vilazodone’s modulatory effects on 5-HT neurons produce a tempered DA release from striatal 5-HT terminals, resulting in an attenuation of hyperactive intracellular signaling, thus allowing a “normalization” of neurophysiological (present study) and molecular [54] activities in these neurons (see also [44]), both critical for avoiding L-DOPA-induced motor abnormalities such as dyskinesia (see [54], for discussion). Future work with cell type-specific experimental manipulations will be necessary to provide more detailed insights into the specific cellular mechanisms underlying the vilazodone effects on striatal neuronal activity.

### 3.4. The Effects of Vilazodone Are Mediated by 5-HTr_1A_

Previous work first indicated that vilazodone, a 5-HTr_1A_ partial agonist [50,52], indeed acts via stimulation of 5-HTr_1A_ to inhibit L-DOPA-induced dyskinesia [53]. Our present results confirm and extend these earlier findings by showing that a selective 5-HTr_1A_ antagonist (WAY-100635) strongly attenuated the antidyskinetic effects of vilazodone for all subtypes of AIMs. Moreover, we investigated the impact of blocking 5-HTr_1A_ on the vilazodone effects on cortical stimulation-evoked MSN activities in dyskinetic animals. Our results show that the ability of vilazodone to ameliorate aberrant corticostriatal signaling was inhibited by the 5-HTr_1A_ antagonist, underscoring the importance of 5-HTr_1A_ for vilazodone’s impact on cellular and behavioral effects. These findings thus provide mechanistic insights into the impact of the serotonergic innervation and 5-HTr_1A_ on corticostriatal activation of MSNs in this dyskinetic PD rat model.

Many 5-HT receptor subtypes, including 5-HTr_1A_, have a fairly wide distribution in the brain [77]. In our study, all drugs, including the 5-HTr_1A_ antagonist, were given systemically, thus precluding conclusions regarding their specific sites of action (receptor location). However, in line with our reasoning, a recent study reported a near-complete suppression in 5-HT neuron firing following administration of another 5-HTr_1A_ agonist (±8-OH-DPAT), an effect that was reversed by WAY-100635 [68]. These findings are consistent with an involvement of the 5-HT transmission to the striatum in vilazodone’s impact on the cellular and behavioral effects of L-DOPA. Future studies using local drug administration will be necessary to ascertain the role of 5-HTr_1A_ on 5-HT neurons in these effects.

## 4. Conclusions

Our results indicate that vilazodone co-treatment has the ability to “normalize” aberrant corticostriatal transmission and striatal circuit activity following repeated L-DOPA administration via modulating 5-HT activity in a 5-HTr_1A_-dependent manner. Vilazodone may help temper L-DOPA-mediated DA input by enabling a more physiological-like release of DA from 5-HT terminals in the striatum. This SSRI/5-HTr_1A_ partial agonist thus appears to be superior to agents acting as SSRIs only or as 5-HTr_1A_ full agonists. Future clinical trials will be necessary to confirm vilazodone’s potential clinical efficacy.

## 5. Materials and Methods

### 5.1. Animals

Adult male Sprague-Dawley rats (225–249 g upon arrival; Harlan, Indianapolis, IN, USA) were housed 2–3 per cage under standard laboratory conditions (12 h light/dark cycle, lights on at 07:00 h; with ad libitum access to food and water). All procedures met the NIH guidelines for the care and use of laboratory animals and were approved by the Rosalind Franklin University Animal Care and Use Committee (protocol # 17-05; approved on 19 April 2017).

### 5.2. 6-OHDA-Induced Dopaminergic Lesions and Stepping Test

Two weeks after arrival, rats received a unilateral 6-OHDA lesion. These lesions were performed as described previously [55,57]. Rats were deeply anesthetized using 2–5% isoflurane vapors (Patterson Veterinary, Greeley, CO, USA). They received an injection of desipramine HCl (20 mg/kg, i.p.; in 0.9% saline; Sigma-Aldrich, St Louis, MO, USA) 30 min prior to 6-OHDA administration. A single unilateral infusion of 6-OHDA HBr (Sigma-Aldrich; 8 µg in 4 µL of 0.9% saline containing 0.1% ascorbic acid) was delivered into the right medial forebrain bundle (coordinates, from bregma: AP −4.3 mm, ML −1.6 mm, DV −8.3 mm; [78]) as previously described [55]. The infusion rate was 0.4 µL/min, and the cannula remained in place for an additional 10 min before being retracted.

The 6-OHDA lesion was assessed by performing a forelimb stepping test [79] pre-surgery and then 4 weeks post-surgery. In this test, the rat is held by an experimenter and moved sideways, with its forelimb on the side opposite to the movement direction touching the bench surface. Normally, the rat will perform adjusting steps during this lateral movement, in our settings, typically 10–14 steps [54,55,57]. Following a >90% DA depletion, the number of adjusting steps with the forelimb contralateral to the lesion drops to three steps or fewer, two to four weeks after the 6-OHDA lesion, while stepping with the forelimb ipsilateral to the lesion is unaffected [55,56]. Only rats that displayed a stepping deficit of three or fewer steps with the contralateral forelimb following the 6-OHDA lesion were selected for this study. The lesion was further characterized by measuring TH immunoreactivity (see below).

### 5.3. Drug Treatments

Starting after a 4-week recovery period, animals with a 6-OHDA lesion that met the inclusion criterion of three or fewer forelimb adjusting steps received drug treatments on five consecutive days/week (Mon–Fri), for two or three weeks. In week 1, all rats received a daily vehicle injection (10% Cremophor EL in 0.9% saline, 2 mL/kg, i.p.; Sigma-Aldrich), followed 30 min later by the L-DOPA (LD) injection (5 mg/kg, i.p., 2 mL/kg; Alfa Aesar, Tewksbury, MA, USA; coadministered with 12.5 mg/kg benserazide HCl; Sigma-Aldrich). In week 2, one cohort received the same treatment of vehicle + L-DOPA as in week 1 (6/Veh/LD; n = 8), and a second cohort received a treatment of vilazodone HCl (VIL) (10 mg/kg, i.p.; Cayman Chemical, Ann Arbor, MI, USA; in 10% Cremophor EL), followed 30 min later by L-DOPA (6/VIL/LD; n = 14).

To assess a potential role for 5-HTr_1A_ in mediating the actions of vilazodone, a third cohort of 6-OHDA-infused rats received vehicle + L-DOPA (6/Veh/LD) in week 1, vilazodone + L-DOPA (6/VIL/LD) in week 2, and in week 3, they received the selective 5-HTr_1A_ antagonist N-[2-[4-(2-methoxyphenyl)-1-piperazinyl]ethyl]-N-2-pyridinyl-cyclo- hexanecarboxamide, 2Z-butenedioate (WAY-100635 (WAY); 0.5 mg/kg, i.p.; Cayman Chemical) 5 min prior to the vilazodone treatment (6/W/VIL/LD; n = 11), in a within-subject design.

### 5.4. Behavioral Analysis

On the second day of each treatment week, a stepping test was performed 60 min after L-DOPA treatment. Dyskinesias were assessed during the last three days of each treatment week (Wed–Fri), using an established and well-characterized rat dyskinesia scale to measure AIMs [64,65]. Briefly, rats were individually placed in clear plastic cylinders, and AIMs were videotaped and their frequency and severity scored during a 1-min period at 30-min intervals, 30 to 180 min after L-DOPA injection. AIMs are classified as axial, limb (forelimb), or orolingual. Their frequency was assessed using the following scale: 0 = absent; 1 = occasional (1 to 29 s); 2 = frequent (30 to 59 s); 3 = continuous but interrupted by external sensory stimuli; and 4 = continuous, not interrupted by strong sensory stimuli) [64].

Additionally, the AIM severity (amplitude) was assessed as follows: Axial AIMs (1 = 30° angle lateral deviation of head and neck; 2 = 30° < angle ≤ 60° lateral deviation of head and neck; 3 = 60° < angle ≤ 90° lateral deviation of head, neck, and upper trunk; 4 = > 90° angle torsion of head, neck, and trunk, often causing the rat to lose balance), forelimb AIMs (1 = minor involuntary movements of the distal forelimb; 2 = low amplitude movements causing translocation of both distal and proximal forelimb; 3 = involuntary movements of the whole limb including shoulder muscles; 4 = strong, ballism-like limb and shoulder movements), and orolingual AIMs (1 = involuntary movements of the orofacial muscles with no tongue protrusion; 2 = involuntary movements of the orofacial muscles with tongue protrusion).

Blinded scorers were allowed to give partial scores such as 0.5, 1.5, 2.5, and 3.5 in order to increase the accuracy of AIM ratings. A severity score for each AIM subtype was calculated by multiplying frequency and amplitude scores for each assessment period (i.e., 30, 60, 90, 120, 150, and 180 min), and these values were added for a total AIM score for each subtype. An overall total AIM score was calculated by adding total axial, limb, and orolingual scores.

### 5.5. In Vivo Single-Unit Electrophysiological Recordings

Electrophysiological recordings were performed after the behavioral studies. All animals were maintained on the same daily treatment regimen as in the last week of their behavioral studies and received their last treatment on the day of the recordings. Thus, MSNs were recorded in rats treated with L-DOPA following an injection of either vehicle (6/Veh/LD), vilazodone (6/VIL/LD), or WAY + vilazodone (6/W/VIL/LD). In each group, several MSNs were recorded before the last drug treatment was received to determine “baseline” responses. Responses after the last drug treatment in these animals are designated “challenge” responses; these were recorded 20–180 min after the last drug administration.

Cortically evoked MSN activity was recorded as previously described [55,59,80,81]. Briefly, rats were deeply anesthetized with urethane (1.5 g/kg in physiological saline), and their temperature was maintained at 37 °C using a heating pad. A bipolar cortical stimulation electrode was implanted ipsilateral to the lesion (coordinates, from bregma: AP +3.0 mm, ML –2.5 mm, DV –1.6 mm; [78]) to target the sensorimotor cortex. Cortical local field potentials on the side contralateral to the lesion (AP +3.0 mm, ML +2.5 mm, DV –1.6 mm) were monitored for the presence of slow, large-amplitude waves to ensure that animals were in a deeply anesthetized state during recordings [59]. Recordings began 1 h after electrode implantation.

Microelectrodes for extracellular recordings were manufactured from 2.0 mm outer diameter borosilicate glass capillary tubing (World Precision Instruments, Sarasota, FL, USA) using a vertical micropipette puller (Narishige, Tokyo, Japan). The microelectrode tip was broken to ~1 µm in diameter by pushing against a glass rod, and the electrode was filled with 2 M NaCl solution. Striatal MSN activity was recorded ipsilateral to the cortical stimulation (and lesion) at the following coordinates: AP 0.0 to +0.75 mm, ML –3.3 to –3.9 mm, DV –3.0 to –6.5 mm. These coordinates targeted the sensorimotor striatum, where the most robust L-DOPA-induced pathophysiological changes occur [54,57,74].

MSN activity was assessed across stimulation trials (50 pulses/trial) by measuring probability and onset latency of action potentials evoked by cortical stimulation at four different current intensities (400 µA, 600 µA, 800 µA, and 1000 µA in separate trials) as described previously [80,82]. The order of cortical stimulation intensity was counterbalanced between cells (i.e., either 400–1000 or 1000–400 µA). Cortically evoked MSN action potentials were amplified (Neuro Data Instruments, Delaware Water Gap, PA, USA), filtered, digitized via a Digidata 1440a (Molecular Devices, San Jose, CA, USA), acquired using Axoscope software (Molecular Devices), and analyzed using Clampfit 10 software (Molecular Devices). Upon the completion of the experiment, rats were quickly perfused with 4% paraformaldehyde, and their brains were extracted for postmortem assessment of DA cell loss by TH immunohistochemistry staining and DA cell counting in the SNc, using stereological techniques (described below).

### 5.6. Tyrosine Hydroxylase Immunohistochemistry

Rat brains were sliced coronally into 50 µm thick sections, using a sliding microtome (SM2010 R, Leica Microsystems, Wetzler, Germany) as previously described [83]. Sections containing the substantia nigra (from bregma: approximately −4.8 to −6.1 mm) were incubated in rabbit anti-TH antibody (1:500; Pel-Freez Biologicals, Rogers, AR, USA) for 24 h followed by a 2-h incubation with biotinylated goat-anti-rabbit secondary antibody (1:200; Vector Laboratories, Burlingame, CA, USA). Sections were then incubated with avidin/biotinylated complex (ABC; Vector Laboratories), and bound complexes were visualized using 3,3′-diaminobenzidine and hydrogen peroxide tablets as previously described [56]. The number of TH-positive neurons was estimated by stereological means (Stereo Investigator, MBF Biosciences, Williston, VT, USA). Briefly, the SNc region in 6 coronal sections (collected at 200 µm intervals) was carefully outlined under 4× magnification using a rat brain atlas [78]. TH+ cells from the SNc on the ipsilateral (lesioned) and contralateral (intact) sides were counted at 100× magnification. Cells were only included if the nucleus and soma were visible and under focus. The extent of the lesion was then calculated as the number of TH+ cells on the lesioned side relative to that on the intact side.

### 5.7. Statistical Analysis

Statistical analysis was performed using GraphPad Prism version 8.0 (GraphPad Software, San Diego, CA, USA). The differences in AIM scores between treatments in within-subject design experiments were assessed using Wilcoxon matched-pairs signed-rank tests, or Friedman ANOVAs, followed by Dunn’s post-hoc tests to identify differences between individual treatments. Stepping scores were compared with two-factor ANOVAs with Tukey post-hoc tests. For electrophysiological recordings, the differences in spike probability and onset latency of cortically evoked responses were assessed using two-factor ANOVAs, followed by Tukey post-hoc tests to describe differences between individual groups. Differences were considered significant if *p* < 0.05.

## Data Availability

The data generated during the current study are available from the corresponding author on reasonable request.
